# Effects of Herb-Partitioned Moxibustion on Autophagy and Immune Activity in the Colon Tissue of Rats with Crohn's Disease

**DOI:** 10.1155/2022/3534874

**Published:** 2022-01-28

**Authors:** Jimeng Zhao, Zhe Ma, Handan Zheng, Yan Huang, Luyi Wu, Huangan Wu, Yin Shi, Huirong Liu, Yanan Liu

**Affiliations:** ^1^Key Laboratory of Acupuncture and Immunological Effects, Shanghai Research Institute of Acupuncture and Meridian, Shanghai University of Traditional Chinese Medicine, Shanghai 200030, China; ^2^Shanghai University of Traditional Chinese Medicine, Shanghai 201203, China

## Abstract

**Objective:**

To investigate the mechanism of action of herb-partitioned moxibustion on CD from the perspective of autophagy and immunity.

**Methods:**

The expression of microtubule-associated protein LC3II and SQSTM1/p62 in the colon tissues was detected by immunohistochemistry. Western blot was used to detect the expression of autophagic and immune-related proteins in the colon, such as LC3II, SQSTM1/p62, Beclin1, ATG16L1, NOD2, IRGM, IL-1*β*, IL-17, and TNF-*β*. mRNA levels of immune factors, such as IL-1*β*, IL-17, and TNF-*β*, and autophagy signaling molecules, such as PI3KC, AKT1, LKB1, and mTOR, were detected by RT-qPCR.

**Results:**

Herb-partitioned moxibustion reduced the protein levels of ATG16L1, NOD2, IRGM, LC3II, and Beclin1 (*P* < 0.01) and both the protein and mRNA levels of IL-1*β*, IL-17, and TNF-*β* in CD rats (*P* < 0.01 or *P* < 0.05), and it also increased the expression of SQSTM1/p62 protein (*P* < 0.01). The modulatory effects of herb-partitioned moxibustion on ATG16L1, NOD2, IRGM, LC3II, TNF-*β*, and IL-17 protein and IL-1*β* protein and mRNA were better than those of mesalazine (*P* < 0.01 or *P* < 0.05). Herb-partitioned moxibustion also reduced colon PI3KC, AKT1, and LKB1 mRNA expressions in CD rats (*P* < 0.01 or *P* < 0.05) and increased mTOR protein expression (*P* < 0.05). And the modulatory effect of herb-partitioned moxibustion on AKT1 mRNA was better than that of mesalazine (*P* < 0.05).

**Conclusion:**

Herb-partitioned moxibustion may inhibit excessively activated autophagy and modulate the expression of immune-related factors by regulating the LKB1-mTOR-PI3KC signal transduction networks, thereby alleviating intestinal inflammation in CD rats.

## 1. Introduction

Crohn's disease (CD) is a complex, chronic, nonspecific inflammatory bowel disease (IBD). CD often presents as segmental skip lesions, is most common in the terminal ileum and adjacent colon, and can affect any part of the digestive tract from the mouth to the anus. The clinical characteristics of CD are mainly abdominal pain, diarrhea, bloody stool, weight loss, fever, and even parenteral manifestations in the joints, skin, eyes, and liver [1]. Epidemiological investigations have shown that the incidence and prevalence of CD show an increasing trend worldwide [2]. Currently, no treatment can fully cure this disease, and clinical treatment strategies are focused on relieving symptoms to prevent complications and retard disease progression. Although many new biological agents have been used for CD [3], they can cause certain side effects and drug resistance, and patients undergoing biological agent treatment are prone to relapse, which has a great impact on their daily life and work.

The interactions of genetic susceptibility, immunity, and environmental factors are important in the pathogenesis of CD. On a certain genetic background, the excessive inflammatory response induced by the immune imbalance of the intestinal host can damage the intestinal tract and its mucosal barrier, which is a key factor affecting the occurrence and development of CD. In recent years, increasing evidence has shown that autophagy-related genetic susceptibility is also closely related to the pathogenesis of CD [4, 5]. Autophagy is a process of intracellular catabolism. It is a common mechanism that exists in the development and aging of organisms to eliminate redundant or damaged intracellular organelles. It is essential for a variety of cellular processes and can be used to maintain biological homeostasis under stress [6]. Autophagy can keep the body's immune tolerance at different levels of adaptive immune response. The activation of autophagy helps to relieve the excessive inflammatory response, and its dysfunction can lead to the occurrence of a variety of inflammation, immune, and metabolic disorders [7]. Under normal conditions, the dynamic balance of *T* helper type 1 (Th1) and Th2 activities maintains the immune homeostasis of the body. Breaking this balance will cause inflammation. In the intestinal mucosa of CD patients, autophagy can induce macrophages to present antigens to Th1 cells through major histocompatibility complex class II molecules, after which Th1 cells secrete interferon-*γ* (IFN-*γ*) and tumor necrosis factor-*β* (TNF-*β*), which in turn promote autophagy in macrophages [8]. Autophagy and inflammatory cytokine actions interact with each other, and abnormal autophagy can break the balance between proinflammatory and anti-inflammatory cytokines, resulting in a severe intestinal inflammatory response and IBD.

Autophagy-related genes such as autophagy-related 16-like 1 (ATG16L1), immunity-related GTP as a family *M* protein (IRGM), and nucleotide-binding oligomerization domain-containing protein 2 (NOD2) have been studied [5]. The genetic variation in these autophagy-related genes is related to the occurrence of CD, and these genes are CD susceptibility genes [9–11]. The key step in the final biological effect of autophagy is the fusion of autophagosomes and lysosomes, leading to the formation of autolysosomes to degrade entrapped cargo materials within autolysosomes, a process collectively called autophagic flux [12]. Heterozygous disruption of the Beclin1 gene in mammals (homolog of yeast Atg6) led to it being the first gene identified in mammals that could induce autophagy [13]. ATG16L1 is a homolog of ATG16 and is mainly involved in the formation of autophagosomes [5]. ATG16L1-mediated autophagy in intestinal epithelial cells plays an important role in the control of intestinal inflammation and TNF-induced apoptosis in colitis [14]. During the process of autophagy, the recognition of ubiquitinated proteins is mediated by the noncovalent interaction between the ubiquitin receptor and ubiquitin through the ubiquitin-binding domain. The ubiquitin-binding protein sequestosome 1 (SQSTM1/p62) is the first reported protein with this adaptor function [15]. SQSTM1/p62 can mediate the autophagic degradation of ubiquitinated proteins and functions as a bridge between microtubule-associated protein 1-light chain 3II (LC3II) and the ubiquitinated substrate to be degraded. SQSTM1/p62 has an important role in the process of autophagic substrate degradation [16].

In addition, a variety of signal transduction pathways are involved in the regulation of autophagy. Many of these signal transduction pathways converge at the target of rapamycin (TOR), which is highly conserved in evolution. This important kinase can regulate the level of autophagy. The mammalian target of the rapamycin (mTOR) signaling pathway plays an important role in the regulation of autophagy. Growth factors regulate mTOR activity mainly through phosphatidylinositol-3-kinase/protein kinase B (PI3K/AKT) signaling pathways, and nutrients and energy inhibit mTOR activity mainly through liver kinase B1/5′-AMP-activated protein kinase (LKB1/AMPK). mTOR is also a key regulator of immune function [17]. Autophagy is involved in the regulation of immune mechanisms in CD via the LKB1-mTOR-PI3KC signal transduction network.

Herb-partitioned moxibustion has definite efficacy in the treatment of CD [18, 19], but its mechanism of action needs to be further elucidated. Using RNA-seq high-throughput sequencing, our preliminary study demonstrated that herb-partitioned moxibustion could regulate autophagy and immune gene expression in CD [20]. Therefore, in this study, we further observed the effect of herb-partitioned moxibustion on the expression of autophagy-related proteins and immune-related factors in CD rat colon tissues. We observed the relationship between herb-partitioned moxibustion and the expression changes of proteins and genes related to the LKB1-mTOR-PI3KC signal transduction network in CD colon tissues to clarify the regulatory effect of herb-partitioned moxibustion on autophagy and immunity in CD. Overall, this study explores the mechanism of the immunological effect of herb-partitioned moxibustion in CD treatment from the perspective of autophagy.

## 2. Methods

### 2.1. Experimental Animals

Adult male Sprague-Dawley (SD) rats, weighing 180 ± 20 g, were provided by the Experimental Animal Center of the Shanghai University of Traditional Chinese Medicine and were purchased from Vital River Laboratory Animal Technology Co. Ltd. (Beijing, China). The license for the use of experimental animals was SCXK (Beijing) 2012-0001. All the animals were housed in a clean grade room with controlled temperature (20 ± 2°C), a light/dark (12 h:12 h) cycle, and 50–70% indoor humidity. All experimental protocols were approved by the Animal Research Ethics Committee of the Shanghai University of Traditional Chinese Medicine (NO. PZSHUTCM211115025).

### 2.2. Preparation of the CD model

The CD models were established by the application of 2,4,6-trinitro-benzene-sulfonic acid (TNBS; Sigma, USA) according to Morris' method [21]. Prior to model establishment, the rats were fasted and given water for 24 h. The rats were weighed, and 2% sodium pentobarbital (30 mg/kg) was administered through intraperitoneal injection (ip). The rats were anally injected with a 5% TNBS solution mixed in 50% alcohol at a 1 : 2 ratio (3 ml/kg) using a rubber tube. All groups of rats received the TNBS injection except for the N and NM groups. The rubber tube was put into the anus 6–8 cm deep. Following the injection, the head of the rat was pushed down for about 1 min to prevent loss of the injected solution. The injection was repeated every 7 days for 4 weeks.

### 2.3. Model Identification

After the experimental CD rat models were established, two rats were respectively selected from normal rats and model rats to ascertain whether the CD model was successfully established by hematoxylin-eosin (HE) staining.

### 2.4. Grouping and Intervention

60 rats were randomly divided into normal control (N) group, CD model control (M) group, model control with herb-partitioned moxibustion (MM) group, normal control with herb-partitioned moxibustion (NM), mesalazine (Western Medicine, Med) group, and normal saline (NS) group, with 10 rats in each group.

After the experimental CD rat models were successfully established, the rats were exposed to different treatments. In the MM and NM groups, the Tianshu (bilateral, ST25) and Qihai (CV6) acupoints were selected [22]. The herbal cake is Chinese medicine powder (*Coptis chinensis*, Radix Aconiti Lateralis, Cortex Cinnamomi, Radix Aucklandiae, Flos Carthami, *Salvia miltiorrhiza*, and *Angelica sinensis*) mixed and stirred with yellow wine to form a thick paste, and the herbal cake was prepared with 1 cm in diameter and 0.45 cm in thickness using a mold. The moxa cone was prepared with 0.6 cm in diameter and height using a mold and its weight was 90 mg. When beginning the treatment, the prepared moxa cone was placed on the top of an herbal cake at the ST25 and CV6 acupoints and ignited. Two moxa cones were used at each acupoint for each treatment once daily for 7 days.

The rats in the Med group were fed with mesalazine (Losan Pharma GmbH, Germany), which was prepared at the proportion of adult and rat of 1 : 0.018 [23], once a day for 7 days.

The rats in the NS group were fed with normal saline, 2 ml per time and once a day for 7 days.

The rats in the N and M groups did not receive any treatment but were grabbed and immobilized using the same method applied to other groups.

### 2.5. Sample Collection

Rats were anesthetized by intraperitoneal injection of 1% pentobarbital sodium (30 mg/kg). After anesthetization, the abdominal cavity was opened and 4–6 cm of distal colon was collected 1 cm from the anus. The colon was divided into three parts: one part was fixed in 10% neutral formalin fixative solution, and the other two parts were placed in cryotubes after cutting into pieces, then frozen in liquid nitrogen for 1 h, and later stored in a −80°C freezer.

### 2.6. Observation of Histopathology and Scoring of the Colon Tissues

Rat colon tissues were fixed in 10% neutral formalin fixative solution for 24 h, dehydrated, embedded in paraffin, sectioned at a thickness of 4 *μ*m, and subjected to HE staining. Histopathological changes of the colon tissues were observed under an optical microscope (BX33, Olympus).

### 2.7. Western Blot Analysis of the Expression of Autophagy- and Immune-Related Proteins in Rat Colon Tissues

The colon tissues of each group stored at −80°C were cut into small pieces, and 100 *μ*l of radio-immune precipitation assay lysis buffer was added to every 20 mg of tissue. After full lysing, the supernatant was collected, and the protein concentration was determined using the bicinchoninic acid assay protein concentration assay kit. The protein extracts were taken, and gel electrophoresis was conducted to obtain the gel plates showing the target proteins. The gel plates were then placed in transfer buffer, and protein bands were transferred to membranes in an electroporator (Bio-Rad, USA) in ice water under a 200 mA constant current for 90 min. After the transfer, the polyvinylidene fluoride membrane was removed, blocked in 5% bovine serum albumin (BSA) at room temperature for 2 h, and washed with Tris-buffered saline with Tween 20 (TBST) on a shaker three times for 5 min each. Primary antibodies against LC3II (CST, USA, 1 : 1000), SQSTM1/p62 (Abcam, UK, 1 : 1000), Beclin1 (CST, USA, 1 : 1000), ATG16L1 (CST, USA, 1 : 2000), NOD2 (Abcam, UK, 1 : 2000), IRGM (CST, US, 1 : 2000), IL-1*β* (CST, US, 1 : 1000), IL-1*β* (Abcam, UK, 1 : 1000), TNF-*β* (BOSTER Biological Technology, Wuhan, China, 1 : 400), and GAPDH (Weiao, 1 : 2000) were added onto the membrane, followed by incubation at 4°C overnight in the incubation box. The membrane was washed with TBST three times for 5 min each. Horseradish peroxidase (HRP) (Biotech Well, Shanghai)-labeled goat anti-rabbit secondary antibody (Jackson 1 : 2000) and HRP-labeled goat anti-mouse secondary antibody (Jackson 1 : 2000) were added and incubated with the membrane at room temperature for 2 h. Then, the membrane was washed with TBST five times for 15 min each. The membrane was reacted with the chemiluminescence detection reagent for 2 min. After that, the membrane was exposed to X-ray film in a dark room, and the X-ray film was developed using appropriate developing and fixing solutions. The film was scanned or photographed, and the molecular weight and net optical density of the target bands were analyzed using a gel image processing system (Bio-Rad, USA).

### 2.8. Detection of mRNAs of Immune Cytokines in Rat Colon Tissue by RT-qPCR

Total RNA was extracted from colon tissue using the Trizol (Invitrogen, USA) method, followed by reverse transcription using a reverse transcription kit (Invitrogen, USA). The reaction system was 0.1% diethyl pyrocarbonate-treated water (8-x) *μ*l, RNase inhibitor (50 U/*µ*l) 0.5 *μ*l, random primers (50pM) 2 *μ*l, and RNA x *μ*l (2 *μ*g). After 65-min water bath treatment, the solution was placed at room temperature for 10 min and centrifuged at high speed for 5 s. The following reagents were added into 1.5 ml centrifuge tubes: RNase inhibitor (50 U/µl) 0.5 *μ*l, 5 × buffer (Invitrogen) 4 *μ*l, dNTPMIX (10 mM/each) 2 *μ*l, dithiothreitol 2 *μ*l, and AMV reverse transcriptase (200 U/µl) 1 *μ*l. The tubes were kept in a water bath at 40°C for 1 h. The solution was treated at 90°C for 5–10 min, then put on ice for 5 min, and centrifuged at high speed for 5s. The prepared cDNA was subjected to PCR amplification. The primer sequences were designed by Primer Express Software v2.0 (ABI, USA), and primers were synthesized by BGI Group. The following were the primer sequences of the targeted genes—GAPDH: forward 5′-GGCAAGTTCAACGGCACAGT-3′, reverse 5′-ATGACATACTCAGCACCGGC-3′; IL-1*β*: forward 5′-CCCAAGCACCTTCTTTTCCT-3′, reverse 5′-TTCATCTCGAAGCCTGCAGT-3′; IL-17: forward 5′-ATCCAGCAAGAGATCCTGGT-3′, reverse 5′- CAATAGAGGAAACGCAGGTG-3'; TNF-*β*: forward 5′-GAAAGCATGATCCGAGATGT-3′, reverse 5′-CAGGA ATGAGAAGAGGCTGA-3′; PI3KC: forward 5′-ATTGCTTTGCCTAAGCACCG-3′, reverse 5′-TGTGGCTATGATTGCCTCCA-3'; AKT1: forward 5′-TTCTACGGTGCGGAGATTGT-3′, reverse 5′-TTATCTTGATGTGCCCGTCC-3'; LKB1: forward 5′-TCAAGGTGGACATCTGGTCA-3′, reverse 5′-CCCGATGTTCTCAAAGAGCT-3'; mTOR: forward 5′-AAAATCCTCATGGTCCGGTC-3′, reverse 5′-TCAAGTTGCCGAGATGGATC-3′. The amplification system was as follows: H2O 5 *μ*l, 2 × SYBR GREEN PCR mix 5 *μ*l, forward primer (10 pM/*µ*l) 1 *μ*l, reverse primer (10 pM/*µ*l) 1 *μ*l, and 1 *μ*l of cDNA. The reaction program was 95°C 2 min, 94°C 10 s, 59°C 10 s, and 72°C 10 s, with a total of 40 cycles. The calculation Ct (target gene) – Ct (internal reference gene) was used to obtain the △Ct value of each gene, the average △Ct value of the model group samples was subtracted from the △Ct value of each experimental group of samples, and this number,△△Ct, was used to calculate 2^−△△^Ct to find the final expression change.

### 2.9. Statistical Methods

SPSS 18.0 software was used for the data analysis. The data were presented as the mean ± standard deviation ($\bar{x}\pm \text{s}$), and the comparison of differences among groups was performed using one-way ANOVA if data conformed to the normal distribution. If the variances were homogeneous, the pairwise comparison was performed using the least significant difference (LSD) test. If the variances of all groups were not homogenous, the nonparametric Kruskal–Wallis test was performed for the analysis. Data that did not conform to the normal distribution were presented as the median and quartiles [*M* (*P*_25_, *P*_75_)], and the rank sum test was performed for the comparison of differences among groups. The significance level of the statistical examination was *β* = 0.05. *P* < 0.05 indicated that the difference had statistical significance.

## 3. Results

### 3.1. Histopathological Observation of Rat Colon in Each Group

**Figure 1:**
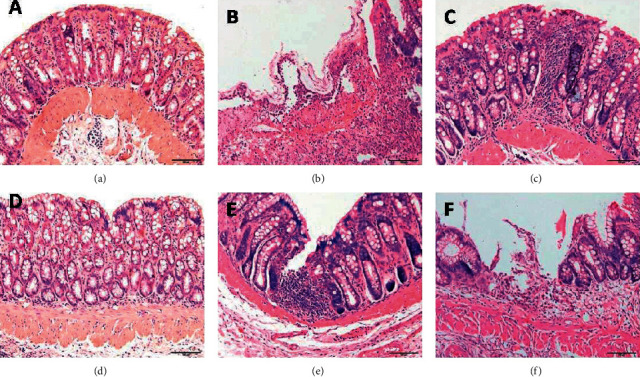
The histological observation of rat colon in each group by HE staining method (200×). (a) normal group; (b) CD model group; (c) CD model with herb-partitioned moxibustion group; (d) normal with herb-partitioned moxibustion group; (e) CD model with mesalazine group; (f) CD model with normal saline group.

As shown in Figure 1, the colon epithelial tissues of the rats in the normal group and the control moxibustion group were intact, with the glands aligned, and no hyperemia, edema, hyperplasia, or ulcers were observed. In the model group, extensive loss of colonic mucosa and the presence of large ulcerations were observed, reaching deep into the muscularis, and many cells on the surface of the ulcer were exuded and were necrotic. However, no tissue repair was seen. In the herb-partitioned moxibustion group, the mucosal epithelium of the colon was relatively intact, and the glands were partially lost, which manifested as healed, shallow ulcers, and many inflammatory cells and fibroblasts were generated. In the mesalazine group, the ulcer of the colon was covered with hyperplastic mucosal epithelium, forming small healed ulcers, atypical hyperplasia of the glands was observed at the edge of the ulcer, and hyperplasia of many fibroblasts and new capillaries was observed in the submucosa. In the saline group, the mucosa of the colon tissue was largely lost, showing a huge ulcer that reached the muscularis. Atypical hyperplasia of glandular epithelial cells, exudation and necrosis of many cells on the surface of the ulcer, and a large amount of granulation at the bottom of the ulcer were observed.

### 3.2. Effect of Herb-Partitioned Moxibustion on the Expression of Autophagy-Related Proteins Implicated in CD

**Figure 2:**
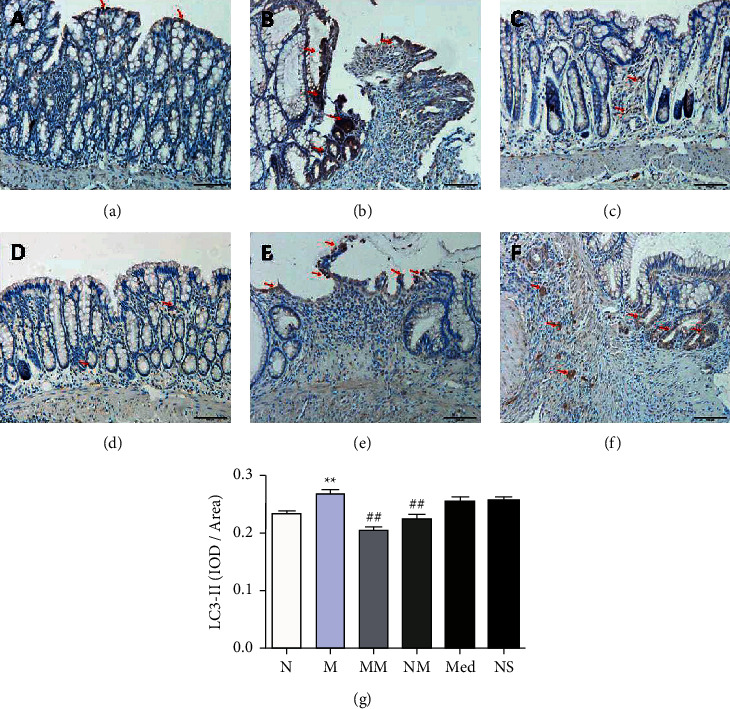
The expression of LC3 protein in colon tissues of rats in each group (200×). (a) normal group; (b) CD model group; (c) CD model with herb-partitioned moxibustion group; (d) normal with herb-partitioned moxibustion group; (e) CD model with mesalazine group; (f) CD model with normal saline group; (g) comparison of LC3 protein expression in colon tissues of rats in each group. ^*∗∗*^*P* < 0.01 vs. N group; ^##^*P* < 0.01 vs. M group. The data are shown as the mean ± SD.

**Figure 3:**
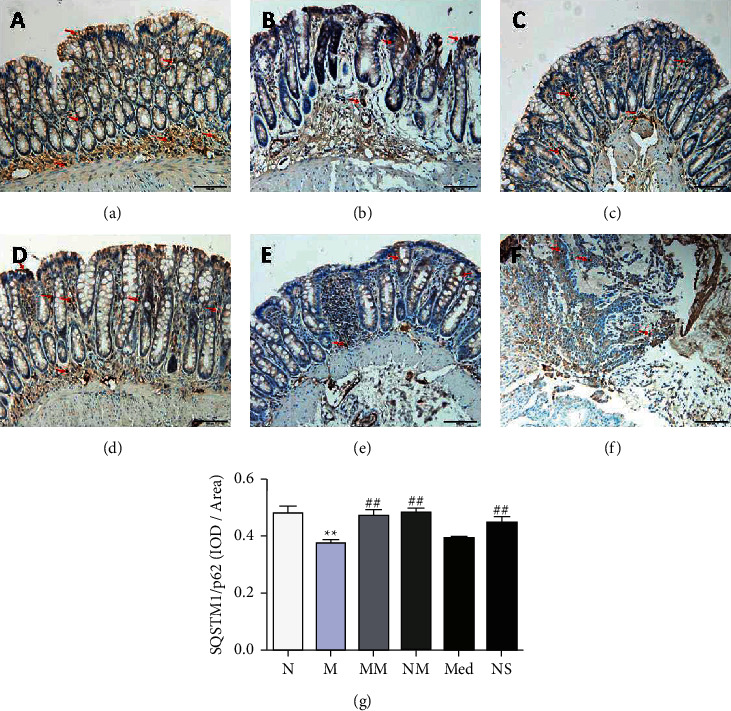
The expression of SQSTM1/p62 protein in colon tissues of rats in each group (200×). (a) normal group; (b) CD model group; (c) CD model with herb-partitioned moxibustion group; (d) normal with herb-partitioned moxibustion group; (e) CD model with Mesalazine group; (f) CD model with normal saline group; (g) comparison of SQSTM1/p62 protein expression in colon tissues of rats in each group. ^*∗∗*^*P* < 0.01 vs. N group; ^##^*P* < 0.01 vs. M group. The data are shown as the mean ± SD.

The expressions of autophagy-related proteins ATG16L1, NOD2, and IRGM were detected. We also detected the proteins LC3II, SQSTM1/p62, and Beclin1, which play key roles in autophagy. The modulation by herb-partitioned moxibustion of these autophagy-related proteins was evaluated. Immunohistochemistry showed that LC3II expression was higher (*P* < 0.01) and SQSTM1/p62 was lower in the rat colon tissue of the CD model than the normal group (*P* < 0.01). LC3II expression was lower (*P* < 0.01) and SQSTM1/p62 was higher in the rat colon tissue of the herb-partitioned moxibustion group than the model group (*P* < 0.01, Figures 2 and 3).

**Figure 4:**
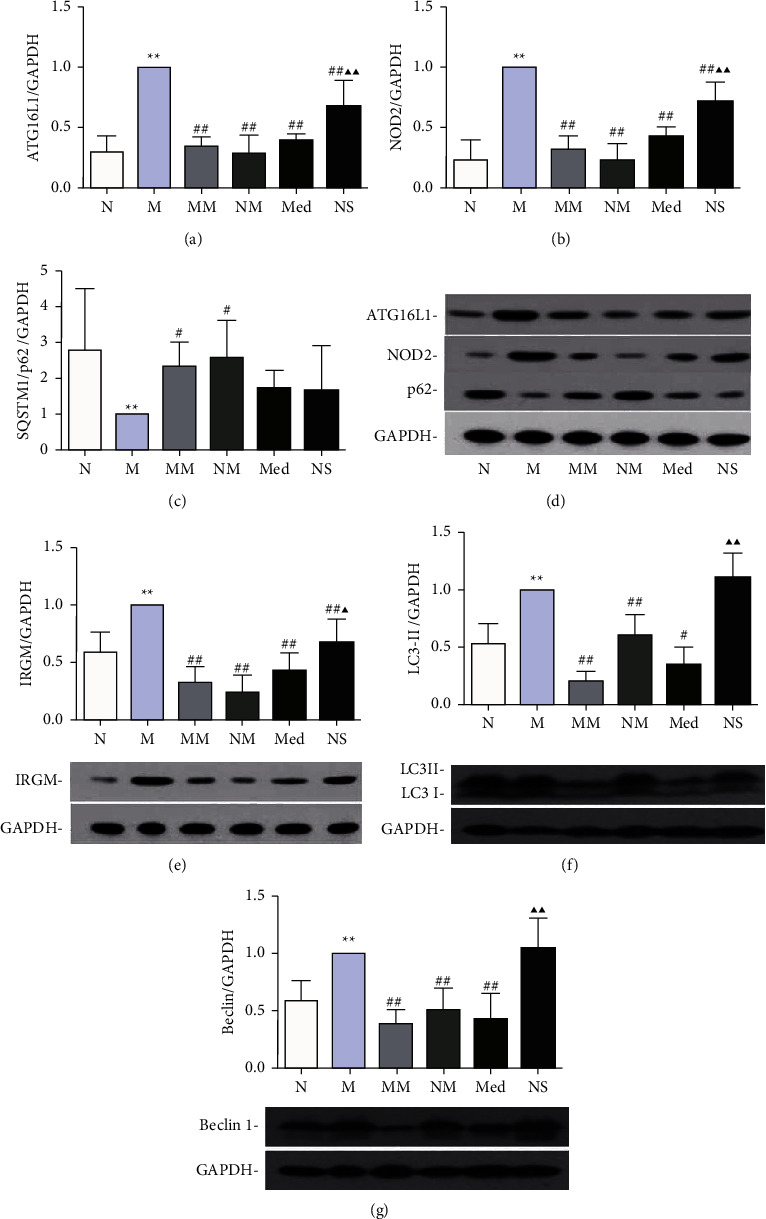
The expression of autophagy-related proteins in colon tissues of rats in each group. (a) expression of ATG16L1 protein in the colon; (b) expression of NOD2 protein in the colon; (c) expression of SQSTM1/p62 protein in the colon; (d) expression of ATG16L1, NOD2, and SQSTM1/p62 proteins in the colon. (e) expression of IRGM protein in the colon; (f) expression of LC3 protein in the colon; and (g) expression of Beclin1 protein in the colon. ^*∗*^*P* < 0.05,  ^*∗∗*^*P* < 0.01 vs. N group; ^#^*P* < 0.05,  ^##^*P* < 0.01 vs. M group; ▲*P* < 0.05, ▲▲*P* < 0.01 vs. Med group. The data are shown as the mean ± SD.

Western blot analysis showed that ATG16L1, NOD2, IRGM, LC3II, and Beclin1 were significantly higher (*P* < 0.01) and SQSTM1/p62 was significantly lower in the CD model group than the normal group (*P* < 0.01). The levels of these six proteins in the colon of the control moxibustion group were not significantly different from those of the normal group. ATG16L1, NOD2, IRGM, LC3II, and Beclin1 in rat colon tissues of the herb-partitioned moxibustion and mesalazine groups were significantly lower than those in the model group (*P* < 0.01 or *P* < 0.05), and the modulatory effects of herb-partitioned moxibustion on ATG16L1, NOD2, IRGM, and LC3II protein expression were stronger than those of mesalazine (*P* < 0.01 or *P* < 0.05). The expression of SQSTM1/p62 protein was significantly increased only in the moxibustion group (*P* < 0.05), and ATG16L1, NOD2, and IRGM proteins were also significantly lower in the saline group (*P* < 0.05) than the model group (Figure 4).

### 3.3. Effect of Herb-Partitioned Moxibustion on the Expression of CD-Related Immune Cytokines

**Figure 5:**
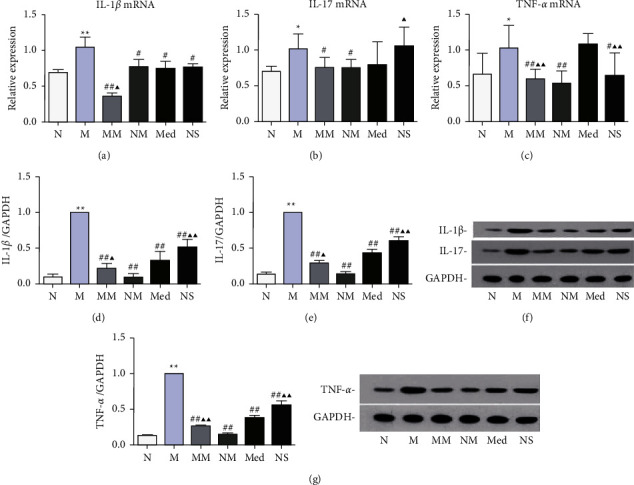
The expression of immune factors in colon tissues of rats in each group. (a–c) mRNA expression of IL-1*β*, IL-17, and TNF-*β* in colon tissues; (d–f) protein expression of IL-1*β* and IL-17 in colon tissues; and (g) protein expression of TNF-*β* in colon tissues. ^*∗*^*P* < 0.05,  ^*∗∗*^*P* < 0.01 vs. N group; ^#^*P* < 0.05,  ^##^*P* < 0.01 vs. M group; ▲*P* < 0.05, ▲▲*P* < 0.01 vs. Med group. The data are shown as the mean ± SD.

Next, we observed the regulatory effect of herb-partitioned moxibustion on the immune-related factors IL-1*β*, IL-17, and TNF-*β* in the CD rat colon tissue. RT-qPCR showed that IL-1*β*, IL-17, and TNF-*β* mRNAs were significantly higher in the model group than the normal group (*P* < 0.01 or *P* < 0.05), and IL-1*β*, IL-17, and TNF-*β* mRNAs in the control moxibustion group were not significantly different from those in the normal group. IL-1*β* mRNA in the herb-partitioned moxibustion, mesalazine, and saline groups was significantly lower than that in the model group (*P* < 0.05), but IL-17 and TNF-*β* mRNA expressions were significantly lower only in the herb-partitioned moxibustion group (*P* < 0.05 and *P* < 0.01, respectively). IL-1*β* mRNA in the mesalazine group was significantly higher than that in the herb-partitioned moxibustion group (*P* < 0.05). Consistent with the RT-qPCR results, Western blot showed that IL-1*β*, IL-17, and TNF-*β* proteins were significantly higher in the model group than the normal group (*P* < 0.01) and did not change significantly in the control moxibustion group. IL-1*β*, IL-17, and TNF-*β* proteins were significantly lower in the herb-partitioned moxibustion, mesalazine, and saline groups than the model group (*P* < 0.01); however, IL-1*β*, IL-17, and TNF-*β* in the mesalazine group were still significantly higher than those in the herb-partitioned moxibustion group (*P* < 0.05 or *P* < 0.01) and significantly lower than those in the saline group (*P* < 0.01, Figure 5).

### 3.4. Effect of Herb-Partitioned Moxibustion on the Activity of the LKB1-mTOR-PI3KC Signal Transduction Network in CD Rat Colon Tissues

The above experimental results showed that herb-partitioned moxibustion could regulate the expression levels of immune-related cytokines and autophagy-related proteins in the CD rat colon tissues, could significantly inhibit the expression of autophagy-related proteins, and could alleviate local inflammation. However, the effective targets and pathways of this regulatory effect needed to be further studied. Since autophagy can participate in the regulation of the immunologic mechanism of CD through the LKB1-mTOR-PI3KC signal transduction network, we further observed the regulatory effect of herb-partitioned moxibustion on the LKB1-mTOR-PI3KC signal transduction network in CD colon tissues to further explain its mechanism of action.

**Figure 6:**
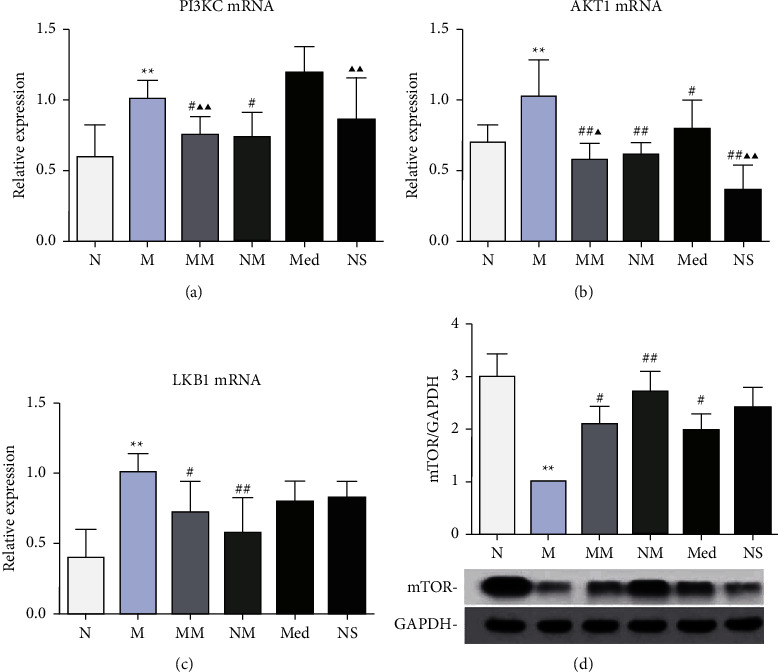
The expression levels of key factors of LKB1-mTOR-PI3KC signal transduction networks in the colon. (a–c) mRNA expression of PI3KC, AKT1, and LKB1 in colon tissues and (d) protein expression of mTOR in colon tissues. ^*∗*^*P* < 0.05,  ^*∗∗*^*P* < 0.01 vs. N group; ^#^*P* < 0.05,  ^##^*P* < 0.01 vs. M group; ▲*P* < 0.05, ▲▲*P* < 0.01 vs. Med group. The data are shown as the mean ± SD.

The results showed that the mRNA levels of PI3KC, AKT1, and LKB1in the rat colon tissues of the model group were significantly greater than those in the normal group (*P* < 0.01). There was no significant difference in PI3KC, AKT1, or LKB1 mRNA expression between the control moxibustion and normal groups. AKT1 mRNA expression was significantly lower in the herb-partitioned moxibustion, mesalazine groups, and normal saline groups than the model group (*P* < 0.01 or *P* < 0.05). Moreover, AKT1 mRNA in the mesalazine group was significantly higher than that in the herb-partitioned moxibustion group (*P* < 0.05). PI3KC and LKB1 mRNAs were significantly lower only in the herb-partitioned moxibustion group (*P* < 0.05). In terms of protein expression, mTOR in rat colon tissues of the model group was significantly lower than that in the normal group (*P* < 0.01), while it was similar between the control moxibustion group and the normal group. Both herb-partitioned moxibustion and mesalazine treatments significantly upregulated mTOR protein in CD rat colon tissues (*P* < 0.05, Figure 6).

## 4. Discussion

For a long time, CD was considered an autoimmune disease controlled by T cell-dependent immune responses. The discovery of ATG16L1, NOD2, and IRGM, autophagy-related genes implicated in CD, reflects the association between inflammation and innate immune gene polymorphism. The ATG16L1 gene is mainly expressed in the colon, small intestine, intestinal epithelial cells, and lymphocytes, and its encoded protein can participate in the metabolism of intracellular autophagosomes and the immune response induced by intracellular pathogens [24]. The single-nucleotide polymorphisms site in the ATG16L1 gene is highly correlated with CD and is an important risk factor for the pathogenesis of CD [25, 26] that can affect intestinal microbes and aggravate the local intestinal inflammatory response in CD [27]. ATG16L1 can also regulate the Nod-dependent cytokine immune response. NOD1 and NOD2, which are closely related to the pathogenesis of CD, can directly enter sites on the bacterial cell membrane with the help of ATG16L1 to initiate autophagy to clear invading pathogens [28, 29]. NOD2 is the earliest discovered gene related to human CD and plays an important role in the intestinal immune system and gut microbiota homeostasis [30]. NOD2 regulates the expression of the proinflammatory cytokines TNF-*β*, IL-1*β*, and IL-6 through the Toll-like receptor pathway and by activating nuclear factor kappa B (NF-*κ*B) [31, 32], playing an important role in innate immunity in IBD. Cooney et al. [33] showed that the NOD2 and ATG16L1 genes together induced the autophagy of dendritic cells in CD, and this process was involved in the NOD2-mediated antigen presentation and bacterial treatment. The protein encoded by the IRGM gene plays an important role in the immunity induced by intracellular pathogens and is involved in the induction of autophagy and the maturation of autophagosomes [34]. IRGM is a key player in mediating p62-dependent selective autophagy of NLRP3 [35]. IRGM can mediate SQSTM1/p62-dependent autophagic degradation through direct interaction with the inflammasome to regulate colon inflammation and protect the body against inflammatory diseases [36]. IRGM/Irgm1 negatively regulates IL-1*β* maturation by suppressing the activation of the NLRP3 inflammasome [35].

Under normal circumstances, autophagy in intestinal epithelial cells can protect tissues against invasion by intestinal pathogens, which is key to the intestinal defense against bacterial invasion [37]. There are abnormalities of autophagy in intestinal cells in CD [38]. The action of TNF-*β* on human and rat colon epithelial cells results in impaired autophagy and loss of the adhesion ability of intestinal epithelial cells [39]. ATG16L1 is not only widely distributed in intestinal epithelial cells but also exists in lymphocytes and macrophages. Together with NOD proteins, ATG16L1 plays multiple roles in innate immunity. On the one hand, autophagy dysfunction in colon epithelial cells cannot effectively remove microorganisms, resulting in their long-term survival in the intestine; on the other hand, abnormal expression of ATG16L1 and IRGM can also cause damage to Paneth cell function and increase the secretion of proinflammatory cytokines [40]. A study on protein-protein interaction found that impaired autophagy can degrade key proteins in Paneth cell function, subsequently affecting the homeostasis of Paneth cells [41]. Therefore, abnormal autophagy in intestinal cells may be one of the mechanisms that cause an excessive inflammatory response in CD intestinal mucosa.

Autophagy is an important component of immune homeostasis. Autophagy and biosynthesis work together to maintain the dynamic balance of cellular macromolecules. Beclin1 is an important autophagy-related protein that is also involved in apoptosis. Many autophagy regulatory proteins directly or indirectly bind to different domains or amino acids of Beclin1 to form protein complexes, thereby regulating autophagy levels and inflammatory responses [42–44]. Beclin1 interacts with the phosphatidylinositol-3-kinase (PI3K) complex and plays a role in regulating autophagy in the trans-Golgi apparatus [45]. LC3II is a mammalian homolog of the yeast autophagy-related protein Atg8 [46] and is the most widely studied molecular marker of autophagosomes. SQSTM1/p62 is also a marker protein of autophagy. The reduction in SQSTM1/p62 expression during autophagy can promote the activation of autophagic flux and regulate the secretion of lipopolysaccharide-induced inflammatory factors under the regulation of the autophagy signaling pathway [47].

The autophagy mechanism can regulate a variety of immune inflammatory responses [48, 49], while immune-related factors can also regulate autophagy. Therefore, cytokines can interact with autophagy signaling pathways. When NF-*κ*B signaling is blocked, TNF-*β* can activate autophagy; the Th1 cytokine IFN-*γ* stimulates autophagy, while the Th2 cytokines IL-4 and IL-13 inhibit autophagy. IL-4 and IL-13 can induce the AKT-TOR signaling cascade, and their inhibition of autophagy can be transformed into the autophagic regulation of intracellular pathogens [50]. Abnormal immune function is one of the main causes of CD onset. IL-17 is a cytokine secreted by Th17 cells that plays an important role in autoimmune diseases and defense responses. IL-17 can bind to its receptor to induce the secretion of chemokines by effector cytokines, thereby promoting the generation and recruitment of neutrophils and macrophages. IL-1*β* and TNF-*β* are important proinflammatory factors. In the onset and progression of CD, they can cause the aggregation and release of inflammatory factors in the intestinal tract; increase the permeability of intestinal epithelial cells; induce inflammation, edema, and the formation of granuloma; and even induce the apoptosis of intestinal epithelial cells [51, 52]. When NF-*κ*B activity is inhibited, IL-1*β* and TNF-*β* can induce autophagy, thereby participating in the regulation of inflammation and infectious diseases.

mTOR, as a key protein kinase, not only participates in the regulation of autophagy by the PI3K/AKT signal pathway in CD but also participates in the regulation of inflammation by the PI3K/AKT pathway. It maintains immune homeostasis through various interactions and plays an important role in IBD [53]. PI3K may also negatively regulate CD autophagy by inducing AKT activation [54]. The PI3K/AKT pathway is activated in CD4+ T cells isolated from the peripheral blood of CD patients, inducing the mTOR signaling pathway and causing the activation of the PTEN (a negative feedback regulatory factor) pathway [55]. The PI3K/AKT signal pathway plays an important role in regulating the release of inflammatory mediators and the proliferation of inflammatory cells. The PI3K-AKT-mTOR-TFEB pathway was activated by advanced oxidation protein products (AOPPs) in macrophages. Inhibition of the PI3K pathway effectively alleviated AOPPs-induced autophagy impairment and M1 polarization both in vitro and in vivo, thus reducing intestinal inflammation in AOPPs-challenged mice [56]. The LKB1 gene is an intracellular serine/threonine kinase that is involved in the regulation of cell proliferation, cell cycle arrest, apoptosis, and energy metabolism by the AMPK and mTOR signaling pathways. The LKB1-AMPK pathway is involved in the regulation of CD autophagy. LKB1 can activate AMPK, subsequently inhibits the expression of mTOR, and induces the activation of autophagy [54]. The CD susceptibility allele of ATG16L1 may be related to the immune synapse formation induced by autophagy and negative regulation of T cell activity. LKB1 is recruited to the immune synapse and induces autophagy through LKB1-AMPK [57]. Therefore, the LKB1-mTOR-PI3KC signal transduction network plays an important role in the regulation of CD autophagy.

In this study, the expression levels of Beclin1 and LC3II proteins in CD rat colon tissues were significantly increased, indicating the activation of autophagy in CD rat colon tissues. SQSTM1/p62 is an important indicator that reflects the dynamic activation process of autophagy, mainly mediating the degradation of autophagosomes. The expression of SQSTM1/p62 protein in the colon of CD rats was significantly lower than that of normal rats, indicating the activation of autophagic flux in the colon tissue of CD rats and the degradation of autophagic substrates. However, herb-partitioned moxibustion and mesalazine can both significantly downregulate the expression of Beclin1 and LC3II proteins in the colon of CD rats. Moreover, herb-partitioned moxibustion can upregulate SQSTM1/p62 protein expression and inhibit autophagy activation in the CD rat colon more strongly than mesalazine. The expression levels of the autophagy-related proteins NOD2, ATG16L1, and IRGM in the CD rat colon tissues were significantly higher than normal rats. However, NOD2, ATG16L1, and IRGM proteins were downregulated in both the herb-partitioned moxibustion and mesalazine groups, indicating that autophagy activity in CD rat colon tissues decreased after herb-partitioned moxibustion or mesalazine treatments. Herb-partitioned moxibustion can also downregulate IL-1*β*, IL-17, and TNF-*β* mRNAs and proteins in CD rat colon tissues, which reflects the correlation between autophagy and immune cytokines. Further observation of the role of the LKB1-mTOR-PI3KC signal transduction network in CD autophagy and the regulatory effect of herb-partitioned moxibustion showed that PI3K, AKT1, and LKB1 mRNAs in CD rat colon tissues were significantly higher than those in the normal group, and mTOR protein was significantly lower than that in the normal group, indicating that key upstream factors of mTOR, including PI3K, AKT1, and LKB1, in the LKB1-mTOR-PI3KC signal transduction network are all activated in CD colon tissues, which could inhibit the expression of mTOR. As a key protein of the LKB1-mTOR-PI3KC signal transduction network, mTOR had decreased activity, and the autophagy switch was turned on. After treatment with herb-partitioned moxibustion, the mRNA expression levels of PI3K, AKT1, and LKB1 in the CD colon tissue were significantly reduced, indicating that herb-partitioned moxibustion can significantly inhibit PI3K, AKT1, and LKB1 expressions, upstream factors of mTOR in the LKB1-mTOR-PI3KC signal transduction network, and promote mTOR expression.

In summary, herb-partitioned moxibustion can significantly inhibit excessively activated autophagy in CD rat colon tissues, regulate the LKB1-mTOR-PI3KC signal transduction network, and downregulate the immune-related factors IL-1*β*, IL-17, and TNF-*β*, thereby alleviating and inhibiting intestinal inflammation in CD rats.

## Data Availability

The initial data used to support the findings of this study are included within the article.
